# Prevalence of increases in functional connectivity in visual, somatosensory and language areas in congenital blindness

**DOI:** 10.3389/fnana.2015.00086

**Published:** 2015-07-01

**Authors:** Lizette Heine, Mohamed A. Bahri, Carlo Cavaliere, Andrea Soddu, Steven Laureys, Maurice Ptito, Ron Kupers

**Affiliations:** ^1^Coma Science Group, Cyclotron Research Center and Neurology Department, University and University Hospital of LiègeLiège, Belgium; ^2^Cyclotron Research Centre, University of LiègeLiège, Belgium; ^3^IRCCS SDN, Istituto di Ricerca Diagnostica e NucleareNaples, Italy; ^4^Physics and Astronomy Department, Brain and Mind Institute, Western UniversityLondon, ON, Canada; ^5^Harland Sanders Chair, School of Optometry, University of MontrealMontreal, QC, Canada; ^6^Brain Research and Integrative Neuroscience Laboratory, Department of Neuroscience and Pharmacology, University of CopenhagenCopenhagen, Denmark; ^7^Laboratory of Neuropsychiatry, Psychiatric Centre Copenhagen and Department of Neuroscience and Pharmacology, University of CopenhagenCopenhagen, Denmark

**Keywords:** congenitally blind, functional connectivity, seed-based analysis, vision

## Abstract

There is ample evidence that congenitally blind individuals rely more strongly on non-visual information compared to sighted controls when interacting with the outside world. Although brain imaging studies indicate that congenitally blind individuals recruit occipital areas when performing various non-visual and cognitive tasks, it remains unclear through which pathways this is accomplished. To address this question, we compared resting state functional connectivity in a group of congenital blind and matched sighted control subjects. We used a seed-based analysis with *a priori* specified regions-of-interest (ROIs) within visual, somato-sensory, auditory and language areas. Between-group comparisons revealed increased functional connectivity *within* both the ventral and the dorsal visual streams in blind participants, whereas connectivity *between* the two streams was reduced. In addition, our data revealed stronger functional connectivity in blind participants between the visual ROIs and areas implicated in language and tactile (Braille) processing such as the inferior frontal gyrus (Broca's area), thalamus, supramarginal gyrus and cerebellum. The observed group differences underscore the extent of the cross-modal reorganization in the brain and the supra-modal function of the occipital cortex in congenitally blind individuals.

## Introduction

The loss of vision from birth causes a myriad of compensatory plastic changes. At the behavioral level, congenitally blind subjects outperform their sighted counterparts in a wide range of non-visual sensory discrimination tasks (Kupers and Ptito, 2014 for a recent review). For example, congenitally blind individuals show improved performance in tactile acuity at the finger tips (Wong et al., 2011) and perform better in pitch discrimination (Wan et al., 2010), syllable recognition (Gougoux et al., 2009) and sound localization (Fieger et al., 2006). Recent behavioral studies also indicate superior abilities in discrimination, identification and awareness of odors (Rosenbluth et al., 2000; Cuevas et al., 2009; Beaulieu-Lefebvre et al., 2011).

Compensatory plasticity is dependent on cross-modal reorganization of the brain in which the occipital cortex becomes recruited by various non-visual inputs (Kupers and Ptito, 2014). Brain imaging studies have highlighted the pivotal role of the visual cortex in the ability of the blind to perform non-visual tasks (Kupers et al., 2011b). Indeed, PET and fMRI studies have reported that congenitally blind individuals recruit their occipital cortex in tasks involving sound and tactile localization (Gougoux et al., 2005; Voss et al., 2008), tactile and auditory motion detection (Poirier et al., 2006; Ptito et al., 2009; Matteau et al., 2010; Sani et al., 2010), spatial navigation (Kupers et al., 2010), odor perception (Kupers et al., 2011a), language (Burton et al., 2003; Bedny et al., 2008, 2011; Striem-Amit et al., 2012) and memory processing (Raz et al., 2005).

Recent neuro-imaging studies also helped to illuminate the question how congenital blindness affects the structural organization of the brain, and through which pathways non-visual information reaches the occipital cortex. Structural brain imaging studies seem to concur that there are significant reductions in gray matter throughout the whole extent of the visual system. These include the optic chiasm, the lateral geniculate nucleus, the posterior pulvinar, and striate and extra-striate visual areas (Pan et al., 2007; Ptito et al., 2008b; Cecchetti et al., 2015). Regions of the ventral visual stream such as the inferior temporal gyrus and the lateral orbital cortex, as well as regions connected to the dorsal visual stream like the hippocampus also show volumetric reductions (Fortin et al., 2008; Jiang et al., 2009). In addition, cortical thickness is increased in the cuneus (Jiang et al., 2009; Kupers et al., 2011a), which is likely due to a reduction in cortical pruning during the early maturation process as a result of lack of visual input, and which may be indicative of alterations in connectivity. White matter changes in the visual pathways include atrophy of the optic tracts and the optic chiasm, reductions of the optic radiations, the splenium of the corpus callosum (Shimony et al., 2006; Pan et al., 2007; Ptito et al., 2008b; Tomaiuolo et al., 2014) and microstructural changes within the ventral visual pathways (Ptito et al., 2008a).

Recent studies have also tried to elucidate functional changes in the blind brain. Brain activation studies (Ptito et al., 2005; Klinge et al., 2010; Sani et al., 2010; Collignon et al., 2013; Ioannides et al., 2013; Kupers and Ptito, 2014) and transcranial magnetic stimulation (TMS) studies (Wittenberg et al., 2004; Kupers et al., 2006) have found evidence for increased functional connectivity of the occipital cortex with auditory and somatosensory areas. Several of the available resting state studies reported stronger connections of the occipital cortex with somatosensory (Watkins et al., 2012) and language areas (Liu et al., 2007; Bedny et al., 2011; Butt et al., 2013; Wang et al., 2013). Other studies, however, concluded that the occipital cortex of the blind has a general reduced connectivity with somatosensory/auditory regions (Yu et al., 2008; Burton et al., 2014), or even larger parts of the brain (Liu et al., 2007; Qin et al., 2014). Some of these differences may be due to small or inhomogeneous study populations, including both congenital and early blind subjects or subjects with and without residual light perception (Butt et al., 2013; Qin et al., 2013; Wang et al., 2013), or to the fact that the resting state scan was acquired after an active functional scanning paradigm (Bedny et al., 2011). To circumvent these issues, we analyzed resting state functional magnetic resonance imaging (rsfMRI) data of a homogeneous group of congenitally blind individuals lacking any residual light perception, using *a priori* defined regions of interest (ROIs) in areas with known roles in visual, somatosensory, auditory and language processing. No task-related functional brain scans were acquired before or after the resting state scans. Using state-of-the-art methods for analyzing rsfMRI data, we mapped out increases as well as decreases in functional connectivity in the congenitally blind brain.

## Materials and methods

### Participants

We included 12 congenitally blind (CB; 5 females, 7 males; age: 42 ± 14 year) and 20 healthy sighted controls (SC; 12 females, 8 males; 42±14 year). Blind and sighted subjects were matched for age, gender, education, and handedness. All our congenitally blind subjects were born blind and had no history of light perception; Table 1 lists their demographics. All participants gave informed consent and the ethics committee of the University of Copenhagen and Frederiksberg had approved the study protocol.

**Table 1:** **Demographics congenitally blind participants**.

**Subject**	**Age**	**Sex**	**Braille (WPM)**	**Cause of blindness**
CB1	59	M	148	ROP
CB2	50	M	75	ROP
CB3	37	F	104	ROP
CB4	63	F	124	ROP, glaucoma
CB5	37	M	100	Unknown eye pathology
CB6	44	M	158	Retinoblastoma
CB7	51	M	75	ROP
CB8	29	F	91	ROP
CB9	28	F	115	ROP
CB10	59	M	130	ROP
CB11	25	F	118	ROP
CB12	27	M	94	ROP

*ROP, retinopathy of prematurity; WPM, words per minute*.

### fMRI data acquisition and analysis

MRI was conducted on a 3T scanner (Siemens Verio) equipped with a standard 32-channel head coil. Functional images were acquired with an EPI sequence (280 volumes, *TR* = 2.15 s, *TE* = 26 ms, flip angle = 78°, *FOV* = 192 mm^2^, 64 × 64 matrix, 43 axial slices of 4 mm). Scan duration was 15 min. Head motion was restricted by placement of comfortable padding around the participant's head. The three initial volumes were discarded to avoid T1 saturation effects. For anatomical reference, a high-resolution T1-weighted image was acquired for each subject (T1-weighted 3D magnetization-prepared rapid gradient echo sequence “3D MP-RAGE”; *TR* = 1.54 s, *TE* = 3.9 ms, *FOV* = 256 × 256 mm, 256 × 256 matrix, 92 slices of 1 mm thickness). Data preprocessing was performed using Statistical Parametric Mapping toolbox (SPM8, Welcome Department of Cognitive Neurology, London, UK) with MATLAB 7.12 (Mathworks Inc., Sherboorn, MA). Preprocessing steps included realignment and adjustment for movement-related effects, slice time correction, co-registration of functional onto structural data, segmentation of structural data, spatial normalization of all data to standard stereotactic Montreal Neurological Institute (MNI) space using the normalization parameters resulted from the segmentation step. Normalized functional data were then smoothed using a Gaussian kernel with an isotropic 8 mm of full-width half-maximum.

Motion correction was applied using an automatic artifact detection tool for global mean and motion outliers (http://www.nitrc.org/projects/artifact_detect/). The groups did not differ significantly in the number of movement artifacted time points (*p* = 0.08). More specifically, one sighted and one congenital blind subject showed movement. Outliers in the global mean signal intensity and motion were identified and included in the subsequent statistical analysis as nuisance parameters (i.e., one regressor per outlier within the first-level general linear models).

Analysis of functional connectivity was done using the connectivity toolbox “Conn,” version 13o (http://www.nitrc.org/projects/conn; Whitfield-Gabrieli and Nieto-Castanon, 2012). An explicit gray matter mask was used. As recently recommended (Behzadi et al., 2007; Murphy et al., 2009; Saad et al., 2012; Wong et al., 2012), we used a regression of nuisance effects before bandpass filtering (RegBP; Hallquist et al., 2013). The data were despiked, and white matter (WM) and cerebrospinal fluid (CSF) components were regressed out as nuisance variables. Noise was regressed out according to the aCompCor method, where the influence of noise is modeled as a voxel-specific linear combination of multiple empirically estimated noise sources by deriving principal components from noise ROIs and by including them as nuisance parameters within the general linear models. This method protects against confounding correlations as produced by other methods, like global signal regression (Murphy et al., 2009; Chai et al., 2012; Saad et al., 2012; Wong et al., 2012). We then applied a linear detrending term. All described steps are part of the standard procedure in the “Conn” toolbox (Behzadi et al., 2007; Whitfield-Gabrieli and Nieto-Castanon, 2012). The residual BOLD time series went through a bandpass filter between 0.008 and 0.1 Hz to reduce the effect of low frequency drifts and high-frequency noise.

**Figure 1:**
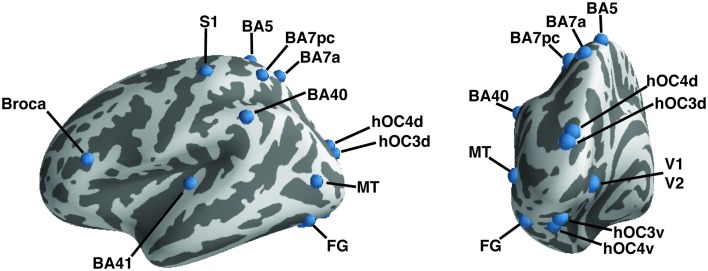
**A priori defined regions of interest**. Regions of interest are shown on the left hemisphere of an inflated brain using PySurfer. Dark areas represent sulci, light gray areas gyri.

Regions of interest (ROIs) were taken from the literature (Geyer et al., 1999, 2000; Amunts et al., 2000; Binkofski et al., 2000; Rademacher et al., 2001; Rottschy et al., 2007; Scheperjans et al., 2008; Caspers et al., 2010, 2013; Kolster et al., 2010; Kujovic et al., 2013); they were defined as 6-mm radius spheres in both hemispheres. We included 15 seeds to assess functional connectivity (Table 2, Figure 1). These seeds were selected within the occipital cortex (i.e., V1, V2, hOC3V, hOC3D, hOC4V, hOC4D, MT/V5, and fusiform gyrus), parietal cortex (S1, lateral BA5, anterior BA7, posterior BA7 and BA40), auditory cortex (A1) and Broca's area.

**Table 2:** **Regions of interest (ROIs)**.

	**Left hemisphere**			**Right hemisphere**			**Literature reference**
	***X***	***Y***	***Z***	***X***	***Y***	***Z***	
**VISUAL AREAS**							
V1 (BA17)	−10	−77	3	20	−73	2	Amunts et al., 2000
V2 (BA18)	−13	−75	6	23	−71	6	Amunts et al., 2000
hOC3d	−15	−97	23	17	−95	24	Kujovic et al., 2013
hOC3v	−20	−88	−3	26	−84	−4	Rottschy et al., 2007
hOC4d	−17	−95	29	19	−94	29	Kujovic et al., 2013
hOC4v	−29	−84	−7	34	−80	−8	Rottschy et al., 2007
hMT (V5)	−48	−75	8	46	−78	6	Kolster et al., 2010
Fusiform gyrus	−30	−76	−9	33	−73	11	Caspers et al., 2013
**SOMATOSENSORY AREAS**							
S1 (BA3b)	−37	−28	55	37	−28	55	Geyer et al., 1999, 2000
BA5	−16	−51	73	13	−55	73	Scheperjans et al., 2008
BA7a	−19	−65	64	20	−65	64	Scheperjans et al., 2008
BA7pc	−34	−53	61	30	−52	61	Scheperjans et al., 2008
BA40 (PF)	−58	−43	39	62	−39	35	Caspers et al., 2008
**LANGUAGE AREAS**							
Broca's area	−42	26	17				Binkofski et al., 2000
**AUDITORY AREAS**							
A1 (BA41)	−42	−21	7	56	−13	8	Rademacher et al., 2001

The fMRI time series from the left and right ROI seeds were averaged and Pearson correlations were calculated between their mean time course and the time course of all other voxels in the brain. Fisher-transformed correlation maps were generated using a general linear model (GLM) to allow for second-level between-group analyses. In all analyses, results were only reported as significant if they survived a height threshold of uncorrected *p* < 0.001 with an extent threshold of FWE-corrected *p* < 0.05 at the cluster level. Significant clusters from the second-level analysis were further examined using SPM. In order to eliminate results derived from a decrease in anti-correlations in blind compared to sighted controls, we used the anti-correlated voxels of the within SC group results as an exclusive mask for the CB > SC comparison. The opposite comparison (i.e., SC > CB) used the anti-correlated mask from the congenitally blind within group analysis. Maps were resliced to the MNI-152 1 mm dimensions using freesurfer and displayed on the FSaverage inflated brain using PySurfer (https://github.com/nipy/PySurfer/).

## Results

### Increased functional connectivity in the blind

**Figure 2:**
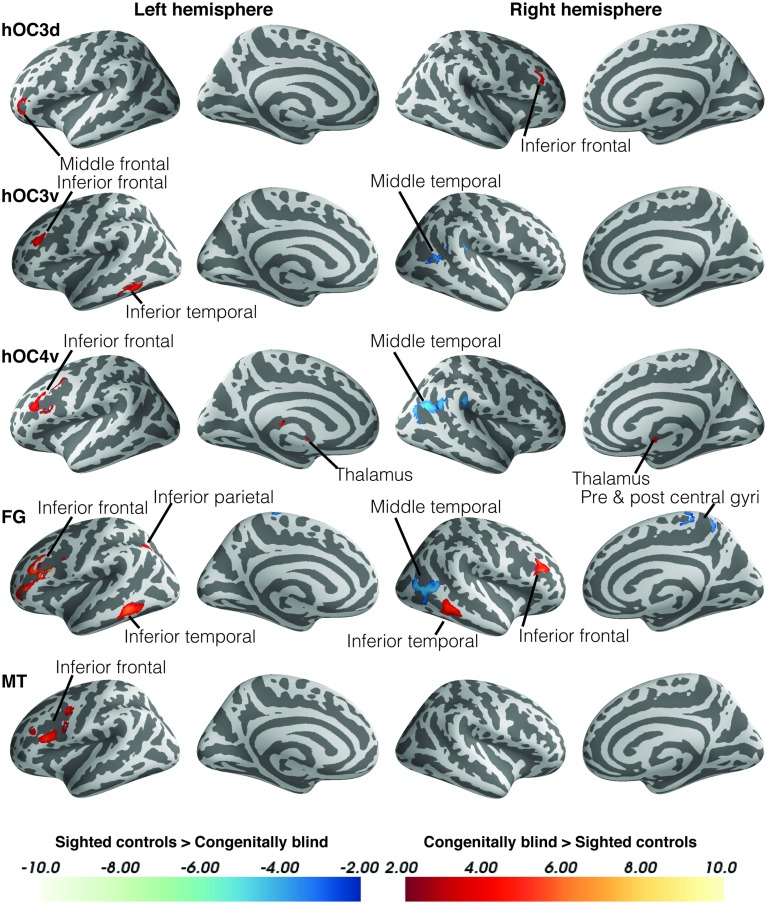
**Differences in resting state functional connectivity between blind and sighted controls (visual ROIs)**. Increases in functional connectivity in the blind group are indicated in red, whereas decreases in functional connectivity compared to controls are shown in blue. Cluster-level FWE-corrected *p* < 0.05. Scale bars indicate *Z*-values. Abbreviations: CB, congenitally blind; SC, sighted controls.

Within-group functional connectivity maps can be found in the Supplementary Material (Figures S1, S2). We found significant group differences in functional connectivity for five out of the eight visual seeds, including hOC3d, hOC3v, hOC4v, fusiform gyrus and hMT+ (Table 3 and Figure 2). One of the most striking results was the increased connectivity between the occipital seeds and Broca's area in the left inferior frontal cortex. More specifically, CB showed an increase in connectivity between hMT+, hOC3d, hOC3v, hOC4v and the fusiform gyrus with the inferior and middle frontal areas, overlapping with BA44 and BA45. The fusiform gyrus and hOC3d also showed increased functional connectivity with the contralateral homolog of Broca's area in the right inferior frontal cortex. Next, connectivity of ventral stream areas hOC3v and fusiform gyrus with the inferior temporal cortex (BA20) was stronger in blind participants. Blind participants also had stronger connectivity patterns between hOC4v and the thalamus, and between hOC3d and hOC4v and the cerebellum. Finally, CB showed increased connectivity between the fusiform gyrus and the inferior parietal cortex and sulcus, which is dorsal to, but not overlapping with Wernicke's area (Figure 2). No group differences in connectivity were observed for V1, V2, and hOC4d.

**Table 3:** **Group differences in functional connectivity (congenitally blind vs. sighted controls)**.

**Seed**		**Brodmann area**	**MNI coordinates (mm)**			**Cluster**	**p-FWE**
			***X***	***Y***	***Z***	**Size**	**Cluster**
**REGION OF INCREASED CONNECTIVITY IN CB**							
hOC3d	Middle cerebellum		0	−60	−38	224	0.0486
	Middle and inferior frontal (R)	BA44,45	52	32	28	275	0.0238
	Inferior frontal and middle frontal (L)	BA45	−44	46	−8	233	0.0427
	Cerebellum (L)		−14	−72	−46	373	0.0067
hOC3v	Inferior frontal (L)	BA44,45	−48	34	26	272	0.0296
	Inferior temporal (L)	BA20	−62	−50	−12	323	0.0155
hOC4v	Thalamus (bilateral)		10	−4	−8	287	0.0193
	Inferior frontal (L)	BA44,45	−50	32	24	454	0.0024
	Cerebellum		2	−80	−16	339	0.0097
FG	Inferior frontal and middle frontal (L)	BA44,45	−40	16	26	989	0
	Inferior temporal (L)	BA20	−56	−46	−10	449	0.003
	Middle and inferior frontal (R)	BA44,45	44	32	20	435	0.0035
	Inferior temporal (R)	BA20	62	−42	−10	381	0.0067
	Inferior parietal (L)	HIP1,2,3, IPC	−32	−58	42	316	0.0149
MT	Inferior frontal (L)	BA44,45	−52	22	22	823	0
BA40	Middle and inferior temporal (R)	MT	48	−60	0	426	0.0026
	V2	BA18	12	−80	42	393	0.0039
BA7pc	Middle cingulate (L)	SPL	−8	−60	58	847	0
	Supramarginal and inferior parietal (L)	BA40	−58	−36	32	221	0.0492
S1	Middle cingulate (R)	BA4,6,SPL	6	−22	46	1213	0
	Supramarginal (L)	BA40, IPC	−50	−32	24	454	0.0016
	Pre and post−central (L)	BA1,2,3,4,6	−46	−20	44	290	0.0143
	Middle and superior temporal (R)	BA22	58	10	−6	233	0.0331
Broca	Middle and inferior occipital (L)	hOC3v, hOC4v	−36	−82	12	1574	0
	Prefrontal (L)	BA10	2	60	10	248	0.0255
**REGION OF DECREASED CONNECTIVITY IN CB**							
hOC3v	Middle temporal (R)	MT,OP,IPC	60	−60	14	371	0.0061
hOC4v	Superior, middle and inferior temporal (R)	MT,OP,IPC	46	−58	12	866	0
FG	Middle temporal (R)	MT	52	−62	4	568	0.0008
	pre and post−central, precuneus	BA4,6	6	−34	58	361	0.0085
BA40	Middle and inferior temporal (R)	BA21	68	−42	−2	344	0.0074
	Middle temporal (L)	BA21	−64	−44	6	328	0.0091
S1	Cerebellum and Vl		10	−78	0	209	0.048
Broca	Inferior frontal (R)	BA44,45	52	8	44	299	0.0121

**Figure 3:**
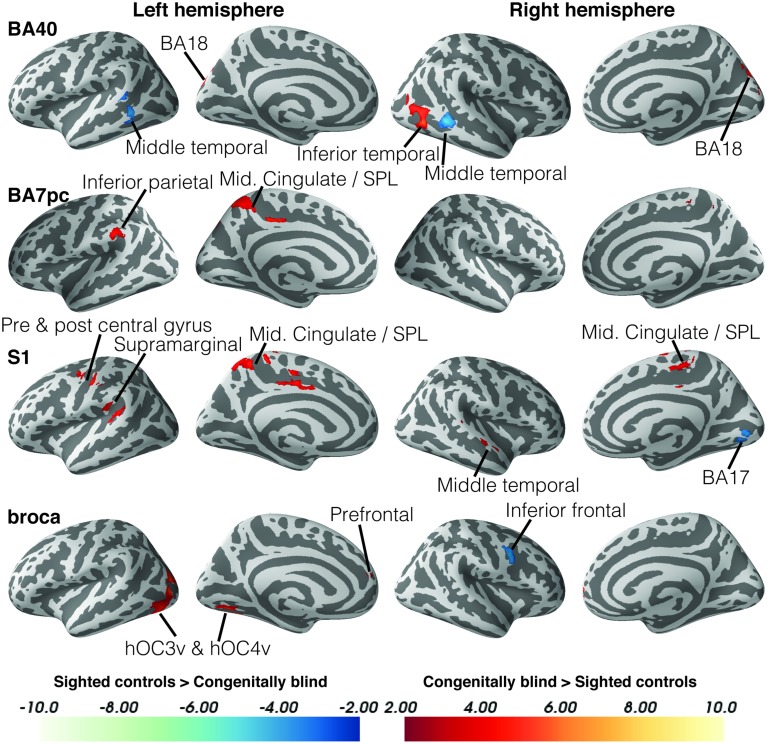
**Differences in resting state functional connectivity between blind and sighted controls (somatosensory and language ROIs)**. Increases in functional connectivity in the blind group are indicated in red, whereas decreases in functional connectivity compared to controls are shown in blue. Cluster-level FWE-corrected *p* < 0.05. Scale bars indicate *Z*-values. Abbreviations: CB, congenitally blind; SC, sighted controls.

Blind individuals also showed increases in functional connectivity for three somatosensory seeds. First, connectivity was increased among somatosensory areas. More specifically, SI and BA7pc showed a stronger connectivity with BA40, and the middle cingulate cortex (left and right respectively). In addition, SI had increased functional connectivity with the primary motor cortex, and middle temporal region (BA22). Our data also revealed increased connectivity between BA40 and the visual areas BA18 and hMT+ (Figure 3).

**Figure 4:**
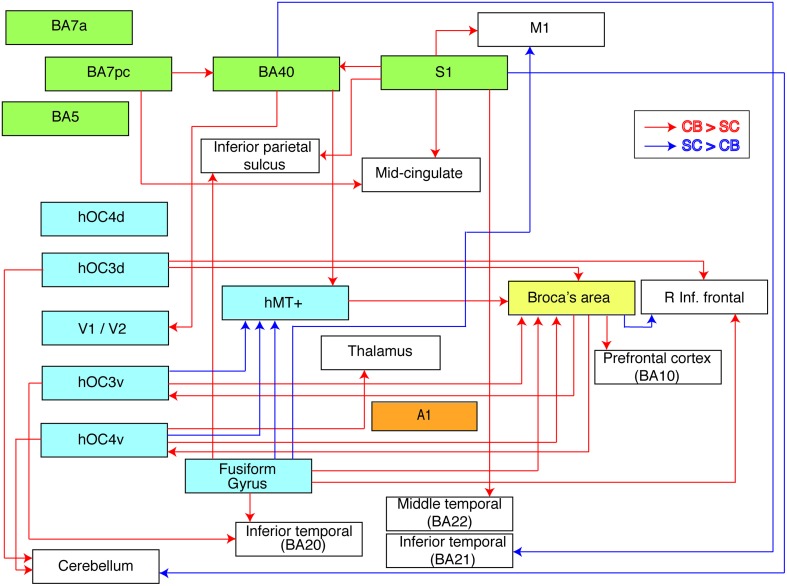
**Flow chart summarizing group differences in functional connectivity**. The used ROIs are represented by colored boxes (blue, visual seeds; green, somatosensory seeds; yellow, language seed; orange, auditory seed). Red arrows indicate an increased connectivity in blind compared to sighted between our seed and the boxes' corresponding brain area. Blue arrows indicate the opposite, i.e., a decreased connectivity in blind compared to sighted individuals.

Broca's area showed an increased functional connectivity with ventral visual stream areas hOC3v and hOC4v, as well as with area BA10 in the left anterior prefrontal cortex (Figure 4). Finally, no significant group differences in functional connectivity were found for the primary or secondary auditory cortex.

### Decreased functional connectivity in blind

Although the largest amount of the observed changes concerned increases in functional connectivity, blind subjects also showed decreases in connectivity in a number of brain areas. More specifically, blind participants showed reduced functional connectivity between ventral visual areas hOC3v, hOC4v and fusiform gyrus and dorsal stream area hMT+ on the one hand, and between fusiform gyrus and MI on the other hand. For the somatosensory seeds, decreases in functional connectivity were observed between BA40 and the inferior temporal area BA21, and between SI and the cerebellum. Blind participants also had a reduced functional connectivity between Broca's area and its contralateral homolog in the right hemisphere.

## Discussion

We investigated alterations in resting state functional connectivity in congenital blindness using a seed-based approach with *a priori* defined ROIs. Although our data revealed a mixture of increases and decreases in functional connectivity in the blind brain, the increases strongly prevailed. The most striking findings of this study were the increases in functional connectivity in the congenital blind brain within the ventral and dorsal visual streams, and between visual cortical regions and Broca's area. In sharp contrast, functional connectivity between dorsal and ventral visual areas was reduced. Figure 4 summarizes the observed increases and decreases in functional connectivity of the congenitally blind brain.

### Increased functional connectivity within the visual streams

Our data show evidence of increased functional connectivity in the ventral visual stream in congenitally blind subjects, more specifically between ventral stream areas hOC3v and fusiform gyrus and the inferior temporal gyrus (BA20). The ventral stream consists of a complex recurrent network between visual areas V1–V4 and the inferior temporal cortex (Kravitz et al., 2013). In sighted subjects, this pathway is implicated in the processing of object quality, object representation or object category (Kravitz et al., 2013). These processes are necessary for object and scene comprehension that form the contents of visual awareness. The fact that this pathway is preserved in blind subjects adds new evidence to the notion that the ventral visual stream holds representations of object shape which are supramodal in nature, and not necessarily visual (Kupers et al., 2011a). For example, non-visual recruitment of the ventral temporal cortex was seen after haptic (Pietrini et al., 2004), non-haptic (Ptito et al., 2012) and auditory (Amedi et al., 2007) exploration of objects in congenital blind subjects.

Congenitally blind subjects also showed increased functional connectivity in the dorsal visual stream, more specifically between BA40 and the secondary visual cortex (V2), as well as between somatosensory areas (BA7pc) and BA40. In normal sighted individuals, the dorsal visual stream is heavily implicated in the visual guidance of action, and consists of a set of projections from the visual cortex to the superior parietal lobule. From there, the dorsal stream splits into the parieto-prefrontal, parieto-medial temporal and parieto-premotor pathway (Kravitz et al., 2011). The parieto-prefrontal pathway connects the parietal cortex to prefrontal regions (e.g., BA46) and is important in top-down control of eye movements and spatial working memory. The parieto-medial temporal pathway, connecting to the posterior cingulate cortex via parahippocampal substructures, is implicated in spatial navigation. Finally, the parieto-premotor pathway connects to premotor regions and is involved in visually-guided actions such as reaching and grasping (Kravitz et al., 2011). Our finding of increased functional connectivity between BA40 and V2, as well as between BA7pc and BA40, are indicative of a fast pathway for information processing from higher order somatosensory to lower level visual areas. This conjecture is in line with results of a recent MEG study indicating that somatosensory information reaches the occipital cortex in the blind via somatosensory and posterior parietal areas (Ioannides et al., 2013), and with results of functional activation studies showing occipital cortex activation following somatosensory stimulation in blind individuals (Ptito et al., 2005). Finally, applying TMS over the occipital cortex can induce tactile sensations in blind subjects trained in the use of a tactile sensory substitution device or in Braille reading (Kupers et al., 2006; Ptito et al., 2008a).

Our results of increased functional connectivity within both the dorsal and ventral visual streams in congenitally blind subjects are in line with results of a recent functional connectivity density mapping study (Qin et al., 2014). Functional activation studies also support the finding of increased connectivity within the dorsal and ventral streams in the blind brain (Kupers et al., 2011a for a review). For instance, congenitally blind subjects trained in the use of the tongue display unit (TDU) showed stronger connectivity between the cuneus and areas within the dorsal and ventral streams (Ptito et al., 2005). In addition, a dynamic causal modeling study showed that the activation of the occipital cortex in blind individuals during an auditory discrimination task is mediated via enhanced corticocortical connections from the auditory to the occipital cortex (Klinge et al., 2010). These functional changes are probably due to anatomical reorganization of the pathways that funnel non-visual information to the visual cortex of the blind (Kupers et al., 2011a). Thus, our rsfMRI data of increased connectivity in the visual streams are supported by results of various functional activation studies showing that the visual streams of the congenitally blind undergo compensatory plasticity and are able to process non-visual information in conjunction with the visual cortex (Dormal et al., 2012; Kupers and Ptito, 2014).

#### Decreased connectivity between the ventral and dorsal visual stream

In sharp contrast with the increase in functional connectivity *within* the visual streams, our data revealed decreases in connectivity *between* the two streams in blind participants. Connectivity of ventral areas hOC3v, hOC4v and fusiform gyrus with dorsal stream area hMT+ was decreased, as well as that between BA40 and the inferior temporal cortex (BA21). There is growing evidence that the dorsal and ventral streams are less independent than originally thought (Schenk and McIntosh, 2010). Although these streams have clear independent functional roles, there is functional and structural evidence that they do not function in an independent manner (Mahon et al., 2007; Borra et al., 2008; Rosa et al., 2009; Schenk and McIntosh, 2010; Zanon et al., 2010). Our data suggest that in the congenitally blind brain the two streams are less interconnected than in the sighted brain. We hypothesize that this may be due to increases in functional connectivity within the two streams. An alternative explanation is that cross-modal non-visual sensory information processing in extrastriate cortex reduces the need for functional connectivity between the streams. Future structural imaging and voxel based morphometric assessment might shed light on the changes in white matter structure within the dorsal and ventral stream to assess whether there is a structural or only functional differentiation between the two streams.

#### Connectivity of the primary visual cortex

We did not find evidence for changes in connectivity in primary visual cortex (V1 and V2). This is in agreement with several other functional connectivity studies (Bedny et al., 2011; Watkins et al., 2012; Butt et al., 2013; Burton et al., 2014). A recent study reported decreased functional connectivity density only in primary visual areas of late blind subjects, while congenitally blind showed increased connectivity between lower tier visual areas and somatosensory areas (Qin et al., 2014), overlapping with the small cluster of increased functional connectivity between BA40 and the primary visual areas observed in this study. However, the literature on changes in functional connectivity of primary visual areas in blind individuals is incongruent. Thus, several fMRI studies reported a correlation between damage to the optic radiation and an event-related fMRI response in visual areas (Seghier et al., 2004), or decreased functional connectivity of primary visual areas with the rest of the brain Liu et al., 2007; Yu et al., 2008; Shu et al., 2009; Wang et al., 2013. These results were explained by the general loss hypothesis. However, this proposed mechanism cannot explain the ubiquitous role of the primary visual cortex in non-visual perceptual and cognitive tasks (Sadato et al., 1996; Amedi et al., 2003, 2004; Burton et al., 2003; Ptito et al., 2005, 2007; Karlen et al., 2006; Voss et al., 2008; Kupers et al., 2010, 2011a; Matteau et al., 2010; Sani et al., 2010; Bedny et al., 2011; Collignon et al., 2011; Watkins et al., 2012). Nor can it explain enhanced effective connectivity with other regions (Wittenberg et al., 2004; Ptito et al., 2005; Klinge et al., 2010). Furthermore, a recent review on structural changes as measured with diffusion concluded that although the literature is inconsistent, it suggest that neither strength nor macro-scale topographic organization is changed in blind individuals (Bock and Fine, 2014). This is congruent with new research showing that functional connectivity based topographic organization of the visual cortices is indistinguishable from sighted controls, and increased functional connectivity to frontal and posterior temporal areas (Striem-amit et al., 2015).

### Visual cortex and language processing

Broca's area (BAs 44 and 45) was the cortical area with the largest amount of alterations in functional connectivity in congenitally blind participants. A total of five visual seeds, hOC3d, hOC3v, hOC4v, hMT+ and fusiform gyrus, showed increased functional connectivity with this area. In addition, Broca's area also showed stronger connectivity with ventral visual areas hOC3v, hOC4v, and with medial prefrontal cortical area BA 10. The current consensus is that the occipital cortex of blind individuals is involved in language processing, showing similar properties as “classical language related areas” (Bedny et al., 2011). Braille reading in blind subjects activates an extensive network of brain areas, including posterior and medial occipital areas, fusiform gyrus, area hMT+, inferior temporal gyrus, inferior frontal, prefrontal, intraparietal sulcus, and somatosensory motor areas (Burton et al., 2002a). More specifically, the increased functional connectivity between visual areas and Broca's area in congenitally blind individuals might relate to the role of the occipital cortex in semantic processing. Whereas semantic processing activates the inferior frontal cortex in both sighted and blind subjects, it activates additionally visual cortical areas in the latter group (Burton et al., 2003; Noppeney et al., 2003; Amedi et al., 2004; Bedny et al., 2011; Watkins et al., 2012). These results expand earlier findings of increased connectivity of the occipital cortex with Broca's area in congenital blindness (Liu et al., 2007; Bedny et al., 2011; Watkins et al., 2012; Butt et al., 2013; Wang et al., 2013; Burton et al., 2014; Deen et al., 2015). The co-activation with Broca's area extends to most of the occipital cortex (Burton et al., 2003; Deen et al., 2015), and might next to language also functionally correlate to working memory (Deen et al., 2015). These results also relate to findings of increased white matter volume within the tracts between prefrontal and occipital areas. More specifically in the fronto-occipital fasciculi (Ptito et al., 2008b; Bock and Fine, 2014).

The increased functional connectivity between Broca's area and hMT+ might be explained by the role of tactile flow processing in Braille reading (Ricciardi et al., 2007). All our congenitally blind were reading braille from when they were children (see Table 1 for speed of braille reading), and Burton et al. (2002a) showed that this area is linked to braille reading only in early bind subjects. Furthermore, the role of the occipital cortex in language processing is further supported by studies showing that rTMS over the mid-occipital cortex not only reduces accuracy of verb-generation (Amedi et al., 2004), but also impairs Braille reading performance (Kupers et al., 2007). Finally, it is worth mentioning that a bilateral occipital stroke in an early blind patient resulted in the loss of Braille reading skills (Hamilton et al., 2000). It is thus interesting for further studies to examine braille performances and related functional connectivity within hMT+ as well as other areas in both congenitally and late blind subjects.

In line with previous results (Bedny et al., 20111), congenitally blind subjects also showed increased functional connectivity between occipital area hOC4v and the thalamus. This finding suggests a thalamo-cortical implication in language processing in the congenitally blind, a conjecture that is supported by the observation that stimulation of left thalamic regions produces language deficits in blind subjects (Johnson and Ojemann, 2000). Our data also revealed a decrease in functional connectivity between Broca's area and its homolog in the right hemisphere. In sighted but not in congenitally blind individuals, the right inferior frontal area is also activated during language tasks (Burton et al., 2002b). Blind subjects might use the visually deprived occipital cortex instead because it is more cost-effective.

### Somatosensory areas

Our results indicate increased functional connectivity between the supramarginal gyrus (BA40) and secondary visual cortex and area hMT+, and between SI and BA40. As stated above, the supramarginal gyrus, occipital, middle temporal and somatosensory cortices are activated by Braille reading (Burton et al., 2002a; Gizewski et al., 2004; Sadato, 2005). We explain the co-activation of somatosensory regions by the tactile input of Braille reading. Indeed, tactile stimuli activate inferior and ventral temporal, as well as somatosensory regions in blind individuals (Pietrini et al., 2004; Ptito et al., 2005, 2012; Matteau et al., 2010; Ricciardi et al., 2013). This co-activation of parietal and visual areas may be at the basis of the superior tactile acuity in blind individuals (Kupers and Ptito, 2014), this might also be related to the increases in white matter volume found in somatosensory and motor areas (Noppeney et al., 2005).

Other rsfMRI studies have reported a decrease of functional connectivity between visual and somatosensory regions (Liu et al., 2007; Yu et al., 2008; Bedny et al., 2011; Qin et al., 2013). However, this finding is at odds with results of several other activation studies indicating strong connectivity between these areas. For instance, functional connectivity was shown to be increased between hMT+ and somatosensory areas (Sani et al., 2010). Furthermore, a recent MEG study from our group revealed activation of the occipital cortex following median nerve stimulation in congenitally blind individuals (Ioannides et al., 2013). A connectivity analysis further suggested that median nerve stimulation first activated primary somatosensory cortex, then the posterior parietal cortex and finally visual areas V3 and V5 (Ioannides et al., 2013). Using somatosensory-evoked potentials, we reported that tactile stimulation of the tongue in blind individuals trained in the use of the tongue display unit first activated the somatosensory cortex and then the occipital cortex (Kupers et al., 2006). Finally, a combined PET-TMS study showed that TMS of the primary somatosensory cortex leads to increased blood flow in the occipital cortex in congenitally blind subjects only (Wittenberg et al., 2004). Together, these findings argue in favor of an enhanced parieto-occipital connectivity in congenital blindness which is supported by the present rsfMRI data.

### Auditory and motor areas

Although many studies have indicated superior auditory abilities in congenitally blind individuals (Kupers and Ptito, 2014), we did not find significant group differences in functional connectivity of auditory areas. Active tasks have indicated stronger cooperation between the auditory and occipital cortices in congenital blindness (Klinge et al., 2010; Collignon et al., 2011). It is possible that in the present study, scanner noise masked a purported increase in resting state functional connectivity between auditory and occipital cortices in blind individuals (De Martino et al., 2014).

We found decreased functional connectivity between the fusiform gyrus and pre- and post-central areas. This is in agreement with several other studies that found decreases between visual areas and motor-related regions, a finding that was explained by the loss of eye-hand coordination in blind subjects (Liu et al., 2007; Yu et al., 2008; Wang et al., 2013). Eye-hand coordination in sighted individuals leads to co-activation of visual and motor areas (Winstein et al., 1997), which is reduced in conditions of congenital blindness.

#### Methodological considerations

Several rsfMRI studies have explored changes in functional connectivity in the blind brain. The reported results are not very consistent and sometimes even conflicting. These differences in results might be due to spurious samples or protocol bias. For instance, some studies included blind subjects with residual light perception (Li et al., 2013; Wang et al., 2013; Burton et al., 2014), or had a mixture of congenitally blind and late-onset blind participants (Butt et al., 2013). Our study cohort was a homogeneous group of congenitally blind participants without any light perception. Furthermore, contrary to some (Liu et al., 2007; Yu et al., 2008), our study used subjects that are not previously used for any analysis, nor was there any active paradigm during the scanning session (Bedny et al., 2011). Another explanation for the inconsistency between studies relates to differences in used methodologies for assessing functional connectivity in rsfMRI data. Early studies used a more exploratory method with atlas-based regions of interest (Liu et al., 2007; Wang et al., 2008; Watkins et al., 2012; Li et al., 2013; Qin et al., 2014), or one or a few hypothesis-driven ROIs, mostly the primary visual area (Yu et al., 2008; Li et al., 2013; Qin et al., 2013). In contrast, our investigation focussed on small areas that are not present in current atlases. Information about the time course (and therefore its functional correlation) of these small areas could also be missed when the time courses of all voxel in an atlas based area are averaged. Our research focused on brain areas with known functional or structural changes in blind subjects in the visual, somatosensory, auditory and language domain, and seed placement was done according to architectonical studies.

We combined the time-series of homologous areas from both hemispheres. For this reason we are unable to draw any conclusions on purported hemispheric differences in functional connectivity. Further, we excluded “increased” or “decreased” correlations in our second level analysis that were caused by anti-correlating time-series in our first level analysis. As with all resting state functional connectivity studies, we are only able to show correlations between different areas, and not any causality. Thereto, DCM or granger causality studies are needed.

## Conclusion

In summary, our data reveal increased functional connectivity *within* both the ventral and the dorsal visual streams in congenitally blind participants. However, connectivity *between* the two visual streams was reduced in blind subjects. In addition, our data revealed stronger functional connectivity in blind participants between the occipital cortex and areas implicated in language and tactile (Braille) processing such as the inferior frontal gyrus (Broca's area), the thalamus, the supramarginal gyrus and the cerebellum. Our results underscore the extent of cross-modal reorganization and the supra-modal function of the occipital cortex in congenitally blind individuals.

## Funding

The Lundbeck Foundation (Denmark), The Danish Medical Research Council, the University Hospital of Liège, the Belgian National Funds for Scientific Research (FRS-FNRS), and the discovery grant from NSERC funded this research.

### Conflict of interest statement

The authors declare that the research was conducted in the absence of any commercial or financial relationships that could be construed as a potential conflict of interest.

## Supplementary material

Figure S1 **Resting state functional connectivity within blind and sighted controls (visual ROIs)**. Within group functional connectivity for sighted controls (left) and congenitally blind (right). Cluster-level FWE-corrected *p* < 0.05. Scale bars indicate *Z*-values.

Figure S2 **Resting state functional connectivity within blind and sighted controls (somatosensory and language ROIs)**. Within group functional connectivity for sighted controls (left) and congenitally blind (right). Cluster-level FWE-corrected *p* < 0.05. Scale bars indicate *Z*-values.
