# Corrigendum: Prevalence of increases in functional connectivity in visual, somatosensory and language areas in congenital blindness

**DOI:** 10.3389/fnana.2015.00106

**Published:** 2015-08-13

**Authors:** Lizette Heine, Mohammed A. Bahri, Carlo Cavaliere, Andrea Soddu, Nina L. Reislev, Steven Laureys, Maurice Ptito, Ron Kupers

**Affiliations:** ^1^Coma Science Group, Cyclotron Research Center and Neurology Department, University and University Hospital of LiègeLiège, Belgium; ^2^Cyclotron Research Center, University of LiègeLiège, Belgium; ^3^SDN Istituto di Ricerca Diagnostica e NucleareNaples, Italy; ^4^Physics and Astronomy Department, Brain and Mind Institute, Western UniversityLondon, ON, Canada; ^5^Danish Research Centre for Magnetic Resonance, Centre for Functional and Diagnostic Imaging and Research, Copenhagen University HospitalHvidovre, Denmark; ^6^Harland Sanders Chair, School of Optometry, University of MontrealMontreal, QC, Canada; ^7^Brain Research and Integrative Neuroscience Laboratory, Department of Neuroscience and Pharmacology, University of CopenhagenCopenhagen, Denmark; ^8^Laboratory of Neuropsychiatry, Psychiatric Centre Copenhagen and Department of Neuroscience and Pharmacology, University of CopenhagenCopenhagen, Denmark

**Keywords:** congenitally blind, functional connectivity, Seed-based analysis, Vision, Resting-state fMRI

The list of authors in the original article has been modified to include Nina L. Reislev and affiliations have been modified accordingly. Dr. Reislev was originally listed in the Acknowledgment section, and has now been added to the list of authors. The Acknowledgment section in the original article has been updated.

Author contributions: RK and MP designed the study. NR recruited participants, collected psychometric and brain imaging data. LH, MB, CC, and AS analyzed the rsfMRI data. LH, RK, and MP wrote the manuscript. SL and AS edited the manuscript and provided scientific expertise.

All the other files remain unchanged. This error does not change the scientific conclusions of the article in any way.

The original article has been updated.

## Conflict of interest statement

The authors declare that the research was conducted in the absence of any commercial or financial relationships that could be construed as a potential conflict of interest.

