# Supplementary figures and images for: Prevalence of increases in functional connectivity in visual, somatosensory and language areas in congenital blindness
Source: Front Neuroanat. 2015 Jul 1;9:86. doi: 10.3389/fnana.2015.00086 (PMC4486836; doi:10.3389/fnana.2015.00086)

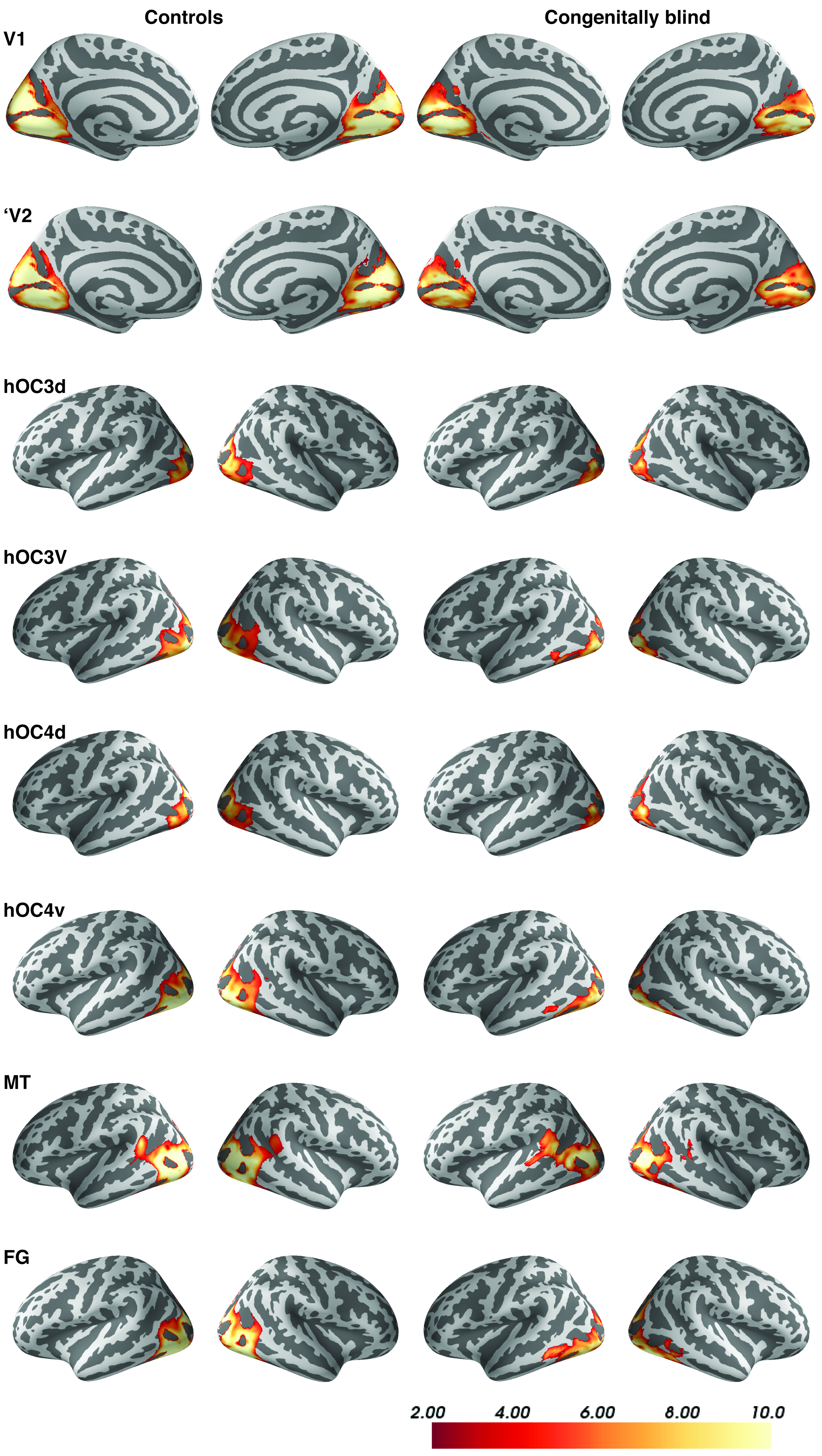

Supplement: Figure S1 — Resting state functional connectivity within blind and sighted controls (visual ROIs). Within group functional connectivity for sighted controls (left) and congenitally blind (right). Cluster-level FWE-corrected p < 0.05. Scale bars indicate Z-values. [file Image1.TIF]

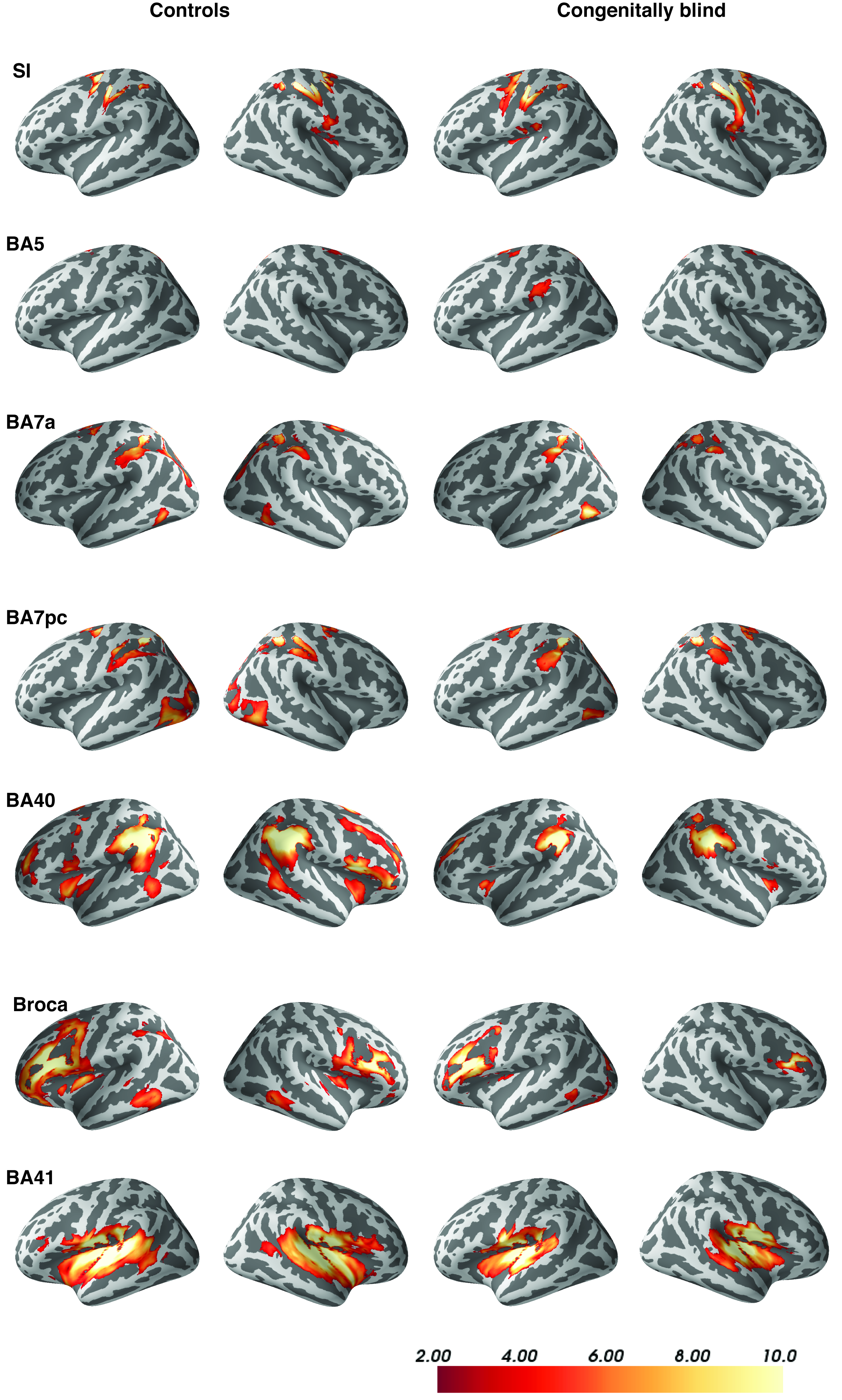

Supplement: Figure S2 — Resting state functional connectivity within blind and sighted controls (somatosensory and language ROIs). Within group functional connectivity for sighted controls (left) and congenitally blind (right). Cluster-level FWE-corrected p < 0.05. Scale bars indicate Z-values. [file Image2.TIF]
